# Encoding mechanical intelligence using ultraprogrammable joints

**DOI:** 10.1126/sciadv.adv2052

**Published:** 2025-04-23

**Authors:** Rui Wu, Luca Girardi, Stefano Mintchev

**Affiliations:** ^1^ Environmental Robotics Laboratory, Department of Environmental Systems Science, ETH Zurich, 8092 Zurich, Switzerland.; ^2^ Swiss Federal Institute for Forest, Snow and Landscape Research (WSL), 8903 Birmensdorf, Switzerland.; ^3^ School of Engineering Mathematics and Technology, University of Bristol, Bristol, UK.

## Abstract

Animal bodies act as physical controllers, with their finely tuned passive mechanical responses physically “encoding” complex movements and environmental interactions. This capability allows animals to perform challenging tasks with minimal muscular or neural activities, a phenomenon known as embodied intelligence. However, realizing such robots remains challenging due to the lack of mechanically intelligent bodies with abundant tunable parameters—such as tunable stiffness—which is a critical factor akin to the programmable parameters of a neural network. We introduce an elastic rolling cam (ERC) with accurately inverse-designable rotational stiffness. The ERC can closely replicate 100,000 randomly generated stiffness profiles in simulation. Prototypes ranging from millimeters to centimeters were manufactured. To illustrate the mechanical intelligence encoded by programming the ERC’s stiffness response, we designed a bipedal robot with optimized ERC passive knees, achieving energy-efficient, open-loop stable walking across uneven terrain. We also demonstrated a quadcopter drone with ERC joints encoding an impact-activated, dual-state morphing.

## Teaser

Robotic joints with highly programmable rigidity enables inverse-designable embodied intelligence across a wide range of scales.

## INTRODUCTION

Nature exploits the nonlinear dynamics of animal bodies to facilitate physical tasks. By fine-tuning mechanical properties such as compliance, inertia, and shape, complex interactions with the environment can be “programmed” in the physical body, enabling operation with minimal neural or muscular input (*1*–*9*). This natural ability to “program” physical responses has inspired the fields of embodied intelligence (*10*–*15*), which aims to simplify and enhance the control of complex interaction tasks such as locomotion (*16*–*29*), grasping, and manipulation (*30*–*35*) by leveraging the mechanical intelligence (MI) of the body.

In nature, MI emerges from the integration of biological tissues with diverse mechanical properties. Biological tissues vary widely in compliance, from the nonlinear softness of muscles and tendons to the rigidity of bones (*36*), which allows evolution to explore a broad range of nonlinear dynamics, embedding diverse functionalities directly in the body. Similarly, a design framework for a programmable robot embodiment should be capable of generating diverse mechanical responses, such as tunable nonlinear stiffness. A rich programmability of this nonlinearity is crucial to MI as it correlates with the complexity of achievable mechanical behaviors (*37*, *38*). A second key requirement for such a framework is inverse design: The mechanical design should be easily derived from a desired nonlinear response of the system. However, to our knowledge, these two essential properties, universal programmability, and inverse designability have not yet been fully realized in robotics. Current robotic MI systems tend to rely on ad hoc solutions, offering limited programmability and lacking robust inverse design methods.

Soft robot materials with hyperelastic or pseudoelastic responses exhibit nonlinear stiffness properties (*39*) but lacks programmability. In contrast, metamaterials allow mechanical nonlinearities to be programmed through morphological design. Local buckling, stretching, and densification within the morphology can induce either softening or hardening effects, promoting abrupt changes in stiffness response (*40*–*44*). To realize inverse design, topological optimization and machine learning methods have been developed to design metamaterials with prescribed stiffness (*45*–*50*). However, these methods are typically hindered by time-consuming finite element simulations, and the accuracy of the achieved stiffness is limited by the inherent uncertainty of the model output. Tensegrity structures also exhibit various programmable behaviors and are considered a pathway toward MI (*51*). It has been shown that a tensegrity’s stiffness can be tailored by adjusting the prestress in the tensile elements (*52*), the property of compressive elements (*53*, *54*), and the alignment of structural elements (*55*). However, these methods typically impose only monotonic effects on the stiffness profile, leading to overall softening and stiffening, thus limited programmability. Moreover, a framework for inverse-designable nonlinear stiffness has yet to be developed. Origami structures, on the other hand, are inherently more designable thanks to the mathematical framework where nonlinear mechanical behavior can be programmed through the folding kinematics. For instance, stiffening can be achieved by panel collisions (*56*, *57*) and curved creases (*58*, *59*) or by inducing long-range intervertex constraints (*60*–*64*), whereas softening has been demonstrated by enabling crease stretching (*65*, *66*), panel stretching (*67*), panel bending (*62*, *68*), and panel shearing (*69*). However, although some of those stiffness-tuning strategies allow inverse designability, the programmability is still fundamentally restricted by the limited versatility of baseline folding patterns (*69*–*72*), and arbitrary nonlinear stiffness responses remain unachievable.

To realize a universally programmable embodiment, we resort to using a simple cam mechanism. Cams have historically been used to encode information for computation and control for over 2000 years (*73*) and have played an important role in early computation devices including mechanical computers and astronomical clocks (*74*–*76*). Cams translate rotational motion into specific actions by encoding mathematical functions or logical operations into their shape. Cams thereby provide a promising foundation for encoding MI. Various spring-loaded cam systems have been demonstrated in specialized mechanisms with controllable stiffness (*77*–*84*). However, a general design framework, combined with a generic, scalable design with mechanical simplicity, remains an open question.

**Fig. 1.**
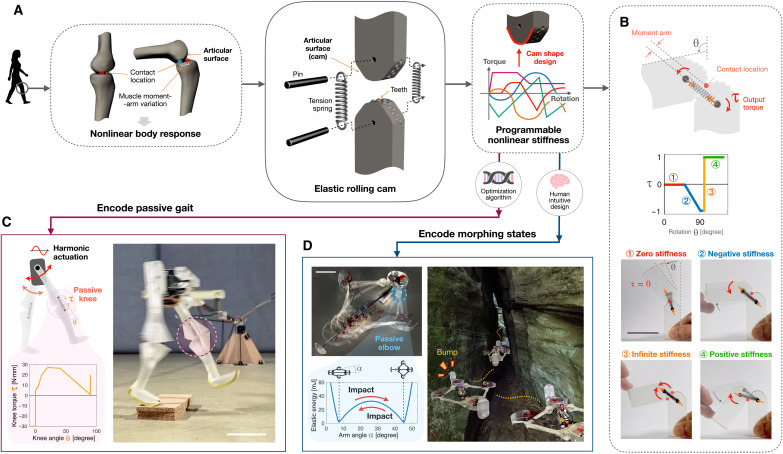
ERC with programmable nonlinear stiffness that encode MI in robots. (**A**) Inspired by bone joints, the ERC uses spring-loaded cams to generate user-defined torque-rotation profiles. (**B**) Illustration of the four basic types of rotational stiffness achieved by the ERC (movie S1). (**C**) ERC knee joint with stiffness optimized for open-loop bipedal walking, achieving stable and robust gait driven by harmonic hip actuation (movie S2). (**D**) ERC elbow joint with bistable stiffness realizes a drone frame that switches configurations upon a gentle impact. Right: Artistic rendering of the drone adapting to fly through a narrow passage by morphing into a narrowed configuration (movie S3). Scale bars, 50 mm.

In this work, we introduce the elastic rolling cam (ERC), a minimalist programmable embodiment with an inverse-designable, multiparameter stiffness response (Fig. 1A). The design is reminiscent of a bone-tendon joint, which leverages the geometry of the articular surfaces to generate a variable muscle moment arm and thereby achieves a nonlinear stiffness response (*85*–*88*). Similarly, the ERC leverages the geometry of the cams to vary the length of a set of springs during rotation, thus altering the spring-stored elastic energy and yielding a tunable torque response. We formulated a comprehensive design framework for selecting appropriate springs and generating cams’ geometry from target torque responses. The basic example shown in Fig. 1B and movie S1 demonstrates four typical types of stiffness responses. Our simulations confirm that the ERC can accurately replicate 100,000 random stiffness profiles (generated by connecting random points in a rotation angle versus torque space), provided that the required spring parameters can be met. To our knowledge, this level of stiffness programmability has not been achieved before.

To showcase the ERC-encoded MI, we have designed a bipedal walker with an ERC-enabled passive knee response optimized by a genetic algorithm. The optimized multiparameter stiffness programmed by the ERC knees allows the robot prototype to walk stably under open-loop harmonic hip actuation across a range of actuation parameters, and over steps, while achieving animal-level energy efficiency (Fig. 1C and movie S2). In addition, we demonstrated dual-state shape morphing in a weight-sensitive quadcopter drone, enabled by ERC elbow joints with bistable response (Fig. 1D and movie S3). Without morphing actuators, this open-loop, impact-activated shape switching allows the drone to fly through narrow passages. We have also demonstrated the ERC’s inherent scalability and structural compliance. These demonstrations highlight the ERC’s capability to serve as a practical pathway toward embodied intelligence, allowing for applications across different scales, safe human interactions, and impact resilience.

## RESULTS

### ERC working principle

The ERC comprises a pair of spring-loaded cams that roll against each other to realize revolute motion. An array of spherical teeth engages to prevent cam slippage. During the revolute motion, the cams’ profile generates a variation of spring length and moment arm—or more fundamentally, the spring-stored elastic energy. This results in a response profile with variable rotational stiffness. The basic example shown in Fig. 1B and movie S1 illustrates how the rolling of the ERC varies the cam’s contact location and spring length, thereby providing various stiffness responses.

### ERC design framework

**Fig. 2.**
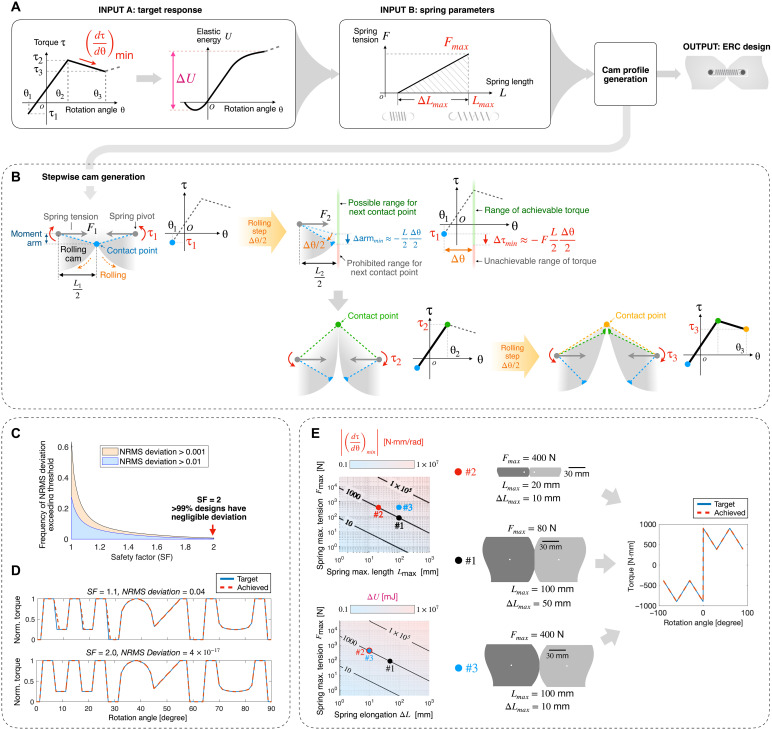
Design framework of the ERC. (**A**) Design process, where spring parameters (input B) are selected based on the target torque response (input A). (**B**) Stepwise cam geometry generation; note that the rotation step Δθ is exaggerated for clarity, and in the real case, $\Delta \theta \ll \theta_{i+1}-\theta_{i}$. (**C**) The SF for spring selection statistically correlates to the accuracy of torque programming. (**D**) A challenging response resembling the letters “ERC” is achieved in simulation with SF=2. (**E**) By adjusting the spring selection, the cam size and geometry can be modified. Left: Springs with different parameters can achieve the same torque reduction rate $\left|{\left(\frac{\mathrm{d\tau}}{\mathrm{d\theta}}\right)}_{\text{min}}\right|$ and energy variation ΔU, plotted with SF=2. Middle: Three ERCs with different springs designed for the same target response (right).

The ERC’s capability to encode MI into robot bodies largely relies on its inverse designability to accurately generate complex, prescribed stiffness responses. We formulated an inverse design framework to derive the ERC’s cam profile from a prescribed stiffness response. As shown in Fig. 2A, the inverse design process requires two inputs: a parameterized target torque response and the selection of spring parameters. Among the three mechanical parameters of a linear-elastic spring—namely, maximum tension force $F_{\text{max}}$, maximum length $L_{\text{max}}$, and maximum elongation $\Delta L_{\text{max}}$—two must be derived from the target response, whereas the third is controlled by the user to influence the final design of the cam. The torque response and spring parameters then feed into a numerical program that computes the cam profile.

The cam profile is generated using a discrete numerical method illustrated in Fig. 2B. We start from one extreme rotational angle and incrementally generate the cam geometry at each rolling step Δθ/2, where the factor of 1/2 accounts for the ERC’s two cams. We incrementally extend the cam shape at each step to create a new contact point that meets the target torque value and spring length, where the spring length is derived from the target energy curve as illustrated in Fig. 2A. To prevent geometrical incompatibility with the previous rolling steps, the position of each new contact point is restricted to the green region shown in Fig. 2B. This thereby limits the maximum torque reduction achievable at each step: $\Delta \tau_{\text{min}}=-F\frac{L}{2}\frac{\Delta \theta }{2}$, where F is the current spring tension, and $\frac{L}{2}\frac{\Delta \theta }{2}$  is the maximum achievable moment arm variation at Δθ/2. Thus, a steeper torque reduction requires higher spring tension or length, and the spring parameters must satisfy a constraint imposed by the target torque reduction rate at each step

$$\frac{F_{\text{max}}\cdot L_{\text{max}}}{4}\geq {\text{SF}}_{1}\cdot \left|{\left(\frac{\Delta \tau }{\Delta \theta }\right)}_{\text{min}}\right|$$ (1)

where $\left|{\left(\frac{\Delta \tau }{\Delta \theta }\right)}_{\text{min}}\right|$ is the maximum torque reduction rate of the target response in N·mm/rad and ${\text{SF}}_{1}\geq 1$ is a user-defined safety factor (SF). When multiple springs are used in an ERC, $F_{\text{max}}$ is the total spring force across all springs. ${\text{SF}}_{1}$ provides a margin that guarantees accurate torque programming. We set ${\text{SF}}_{1}=2$ by default according to our statistical analysis discussed later in this section.

In addition, because the joint’s elastic energy originates solely from the spring, the spring elastic energy capacity—the maximum elastic energy that the spring can store—must exceed the target energy variation ΔU (defined in Fig. 2A). Therefore, we require the spring to satisfy a second constraint

$$\frac{F_{\text{max}}\cdot \Delta L}{2}\geq {\text{SF}}_{2}\cdot \Delta U$$ (2)

where ΔU is the maximum elastic energy variation required by the target response and $\frac{F_{\text{max}}\cdot \Delta L}{2}=U_{\text{spring}}$ is the spring energy capacity in mJ (assuming that the spring is linear elastic). A linear-elastic spring has zero tension when its elastic energy is depleted; thus, a higher ${\text{SF}}_{2}$ prevents the spring from reaching a low tension force, which guarantees the ERC’s accurate torque programming.

For simplicity, we introduce a unified SF: $\text{SF}=\text{min}\left({\text{SF}}_{1},{\text{SF}}_{2}\right)$. We used a Monte Carlo approach to analyze the effect of SF by simulating ERC designs with 100,000 randomly generated target stiffness responses and springs (details about the statistical analysis are available in Materials and Methods). Torque accuracy was assessed by evaluating the normalized root mean square (NRMS) deviation (normalized by the peak torque variation, maximum torque − minimum torque) between the simulated ERC responses and the design targets. We used a Monte Carlo simulation because the ERC profile is generated through a stepwise numerical method, and a straightforward analytical programmability analysis is not feasible. Here, the programmability refers to the design method’s capability to accurately achieve any arbitrary target torque response. The result is shown in Fig. 2C. It can be seen that a large SF leads to a more accurate torque response, and with SF=2, over 99% of the simulated designs have NRMS deviation below 0.1%. An example is given in Fig. 2D, where a challenging stiffness profile resembling the letters “ERC” is replicated with negligible deviation SF=2.

We thereby set a default SF of 2 and inverse design the ERC through the process illustrated in Fig. 2A. A Matlab program is developed to implement this design process. The design process enables the user to adjust one of the three spring parameters to directly influence the final geometry of the cam. As shown in Fig. 2E, the first constraint from torque reduction rate creates a trade-off between spring tension and length, affecting the overall size of the ERC (as the spring length equals the distance between spring pivot points on the cams). This allows the same target torque response to be achieved with a stronger spring paired with a smaller cam, as illustrated by the designs #1 and #2 in Fig. 2E. The second constraint from energy introduces a trade-off between spring tension and elongation and changing the spring tension leads to cam shapes that vary in their circularity, as illustrated by the designs #1 and #3 in Fig. 2E.

### Demonstration of stiffness programmability

**Fig. 3.**
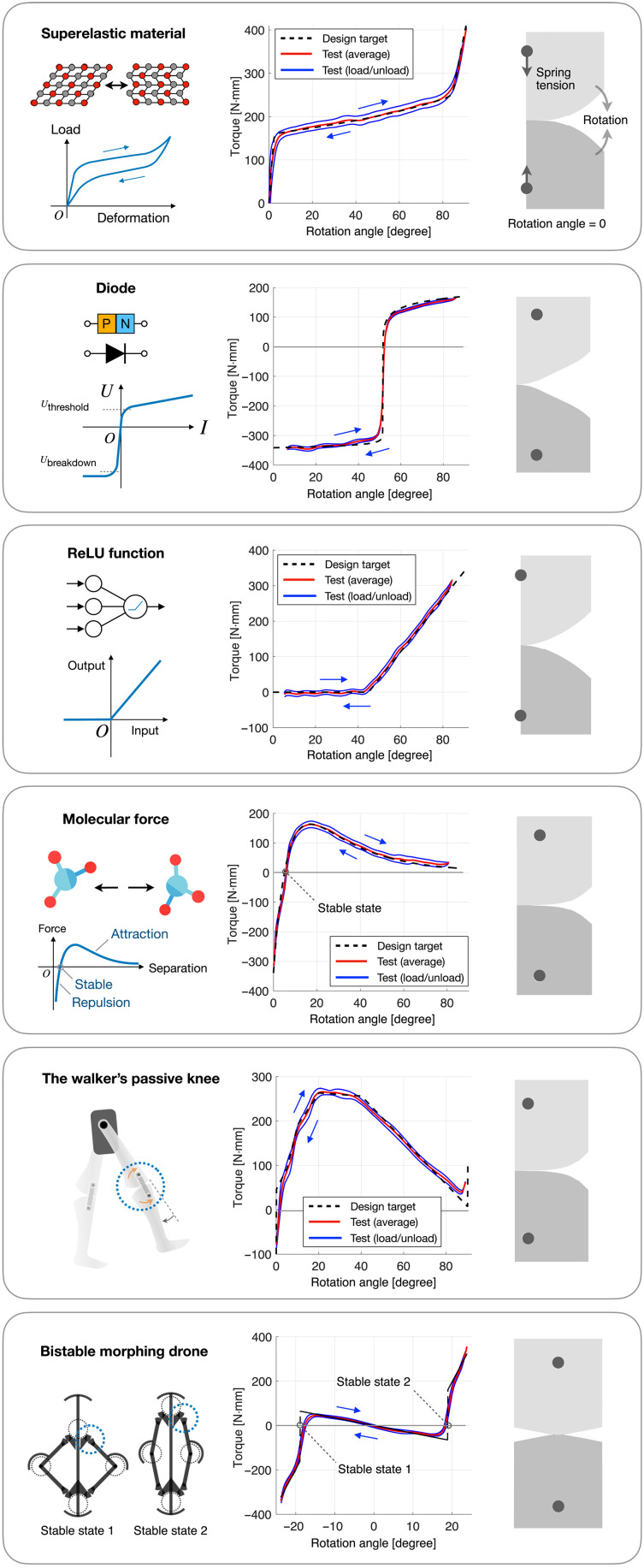
Stiffness programming of the ERC. (**Left**) Various systems that the ERC is designed to replicate. (**Middle**) Experimental stiffness response, including the original loading and unloading curve (blue), and their average (red), plotted against the design target (black dashed line). (**Right**) Geometry of the ERC, illustrated at 0° rotation angle.

To experimentally validate the ERC design, we manufactured prototypes with six different stiffness responses as illustrated in Fig. 3. The six responses are selected because they exemplify several basic types of mechanical responses that are potentially useful in robotics. These include (i) the superelastic response typical of shape memory materials (*89*) and mechanical metamaterials (*44*, *65*, *90*), which exhibits a load-adapting behavior that switches from high stiffness to high compliance upon a critical external load—ideal for compliant robotic systems requiring both rigidity and flexibility; (ii) the exponential diode response with two thresholds, which can potentially realize mechanical signal rectification and logic gates; (iii) the Rectified Linear Unit (ReLU) function, commonly used as activation function in machine learning, potentially useful for MI; (iv) the molecular force profile, mimicking the monostable behavior that stabilizes systems of molecules or potentially robots in highly dynamic environment; (v) the robotic knee joint response, optimized for bipedal walking stability and used in our demonstration (see the section Encoding bipedal walking gait in passive knees); and (vi) the bistable robotic joint response, designed for our dual-state morphing drone demonstration (see the section Encoding dual-state morphing in bistable elbow joints).

We measured the torque responses of the six ERCs using a setup shown in fig. S1. Test results show that the ERCs can follow prescribed stiffness responses. To quantify the deviation between the average test result and the design target, we normalize the responses by the peak torque variation (maximum torque − minimum torque) and evaluate the NRMS deviations. The six ERCs (from top to bottom in Fig. 3) have NRMS deviations of 1.2, 3.5, 1.6, 1.9, 3.0, and 4.1%. The deviation is most noticeable where torque changes sharply. Potential causes of these deviations include rolling cams diverging from the ideal point-contact scenario due to local deformations of the cam, which dampens torque variations by hindering the movement of contact point, frictional interactions between teeth causing fluctuations in the response, and limited accuracy in manufacturing and alignment of the cams. Details about the prototypes’ design, manufacturing, and testing can be found in Materials and Methods and in Supplementary Text.

### Scalability

**Fig. 4.**
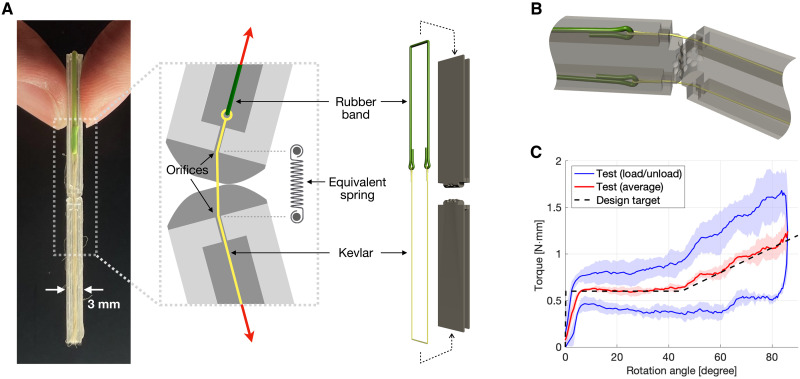
Scaled-down millimeter-sized ERC. (**A**) Photo of the prototype, along with schematics of the operating principle and design. (**B**) Translucent three-dimensional rendering of the ERC model, showing the layout of rubber band and Kevlar fiber. (**C**) Experimentally measured stiffness response of the millimeter-sized ERC averaged from three samples, including their original loading and unloading curve (blue), their overall average (red), the SD (shaded), and the design target (black dashed line).

The size of an ERC depends on the spring length (which equals the distance between the spring pivot points on the cams). As discussed earlier and illustrated in Fig. 2E, the choice of spring length offers design scalability by allowing the trade-off between the spring length (thus ERC size) and tension. Meanwhile, the ERC is suitable for miniaturized robots because such robots typically demand lower torque and thereby lower $|{\left(\mathrm{d\tau}/\mathrm{d\theta}\right)}_{\text{min}}|$ and ΔU, thus requiring shorter springs with lower tension that are suitable for small ERCs. To demonstrate such potential, we designed a millimeter-scale ERC, depicted in Fig. 4, which has a mass below 1 g, and a cam width of 3 mm—one order of magnitude smaller than the centimeter-scale prototypes tested in Fig. 3. At such a scale, elastomers provide a more practical form of elasticity than metallic springs; thus, rubber band is used. As illustrated in Fig. 4 (B and C), the rubber band pulls on a Kevlar fiber yarn that passes through orifices to replicate the effect of a pulling spring with linear elasticity. Test results, plotted in Fig. 4D, show that the ERC provides the prescribed response, whereas the hysteresis is larger than the centimeter-scale models due to friction between the Kevlar fiber and the orifices. Because of the greater influence of manufacturing defects at a smaller scale, as well as the uncertainties in friction, the scaled-down ERC has less accurate torque response than the larger system. Further downscaling is still theoretically feasible although beyond the capability of our manufacturing method. The ERC can also be scaled up for applications in larger systems. Additional details about manufacturing and testing are included in Materials and Methods.

### Encoding bipedal walking gait in passive knees

In this case study, we show that the ERC can provide programmability to an underactuated bipedal robot with an ERC knee joint. By leveraging multiparameter nonlinear knee stiffness, the robot achieves a stable, robust, and energy-efficient gait that adapts to a range of actuation parameters and uneven terrain (see movie S2). This contrasts with the traditional “passive walkers” that typically lack practicality due to their limited robustness and narrow operation window, resulting from the insufficient tunability of their bodies and thus their embodied intelligence (*16*, *17*).

**Fig. 5.**
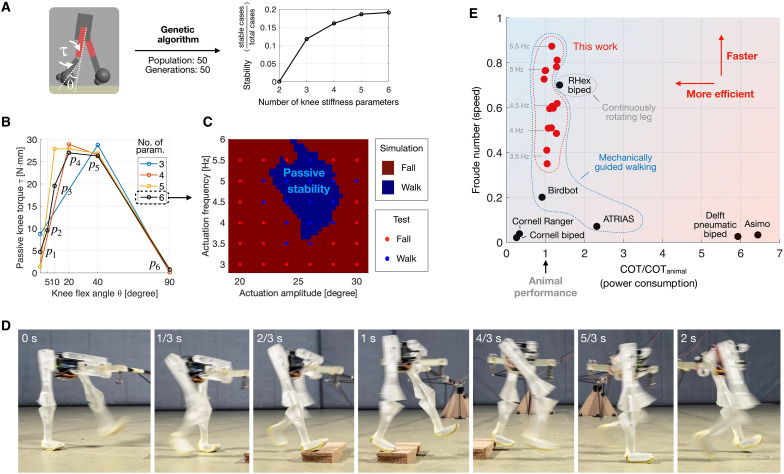
Stable and efficient bipedal walking encoded by ERC knee joint with optimized stiffness parameters. (**A**) We optimized the passive knee stiffness with the objective of stable walking under a widest possible range of harmonic hip actuation frequencies and amplitudes, where higher knee programmability allows better MI-stabilized walking, as evidenced by the increased stability [relative area of the blue region in (C)] with larger numbers of programmable parameters. (**B**) Optimized knee stiffness responses with three to six parameters. (**C**) Stability map showing both simulation (shade) and experimental (dots) results from the six-parameter knee. (**D**) Prototype walking across a step, where the robot is constrained in the sagittal plane and allowed to pitch. (**E**) Performance comparison with existing bipedal robots, where our design has reached a power efficiency close to animals and a relatively fast walking with speed adjustable by hip actuation frequency.

Our bipedal robot uses a minimalist design, with each leg having two degrees of freedom: a passive one from the ERC knee joint and an active one at the hip joint driven by a harmonic hip actuation at prescribed frequencies and amplitudes. In the simulation environment (further explained in Materials and Methods), the robot’s movement is constrained in the sagittal plane and is free to pitch. The knee stiffness profile is then optimized for a robust walking—i.e., stable walking under a wide range of actuation frequencies and amplitudes—using a genetic algorithm. To showcase the impact of body programmability on MI-empowered robots, we optimized the robot with various numbers of knee stiffness parameters: from a basic two-parameter response replicable with a preloaded linear spring to a more complex six-parameter response achievable by the ERC, where the parameters $p_{1}-p_{6}$ (as defined in Fig. 5B) can take arbitrary values between 0 and 60 N·mm. We avoided higher stiffness values to prevent overly stiff legs that restrict knee flexion. In Fig. 5A, the successful walking rate (area fraction of the blue region in Fig. 5C) is plotted against the number of knee stiffness parameters. It can be seen that, even after the same optimization process, the two-parameter knee response failed to provide any stable gait, whereas the six-parameter response achieved stability under a broader range of actuation parameters. This outcome underscores that a larger number of stiffness parameters or a higher body programmability enables more capable embodied intelligence control. Details about the simulation and optimization are included in Materials and Methods.

A prototype is manufactured to experimentally verify the stability (fig. S2A). During the tests, the robot is constrained within its sagittal plane using a pivoted boom (fig. S2B). The boom has a weak torsional stiffness to allow the pitching motion during walking and fall. The test results are plotted in Fig. 5C, showing stability across a range of harmonic hip actuation. Furthermore, the robot has achieved robust walking across a step of 15 mm, or 10% of its hip height, as seen in Fig. 1D and movie S2. Further details about prototype construction and testing are included in Materials and Methods.

Besides stability, we also characterized the robot’s performance regarding energy efficiency and walking speed. We used the cost of transport (COT) as a dimensionless efficiency metric (*26*): COT=P/mgv, where P is the power consumption in watts, m is the robot’s weight (0.27 kg in our case), g is the gravitational acceleration (9.81 m/s^2^), and v is the average speed in m/s. To characterize the speed, we used the dimensionless Froude number (Fr) (*26*): $\text{Fr}=v^{2}/\mathrm{gl}$, where l is the standing hip height (0.156 m in our case). We compared the metrics with various bipedal robots from the literature (*16*, *26*), and the results are plotted in Fig. 5E, where the horizontal axis denotes the COT ratio between robots and typical animals, COT/COT_animal_. Here, COT_animal_ is estimated based on the COT of running terrestrial birds with the same body mass as the compared robot, using an allometric relationship described in the literature (*26*). It is worth noting that three robots, including ours, have mechanical guidance systems (e.g., the boom system shown in fig. S2) that provide stability in certain directions, likely reducing power consumption. The RHex has wheel-like, continuously rotating legs that tend to enhance speed and efficiency. It can be seen that our robot has reached animal-level efficiency while achieving fast walking according to the Froude number (Fr). The walking speed can be adjusted by changing the frequency of hip harmonic actuation: With the frequency increasing from 3.5 to 5.5 Hz, the speed varied from 0.73 to 1.16 m/s.

These results provide insights into the potential application of ERCs in joints of legged robots, where the passive stiffness responses can be used to enhance the robustness and energy efficiency of walking or to off-load active control effort to MI.

### Encoding dual-state morphing in bistable elbow joints

Recently, studies on morphing quadcopters have begun to emerge, where the drone’s body changes shape—either through closed-loop actuation or passive adaptation—to assist tasks including negotiating narrow passages, executing fast maneuvers, performing aerial manipulation, etc. (*24*, *25*, *91*–*95*). Quadcopters, as a typical type of weight-sensitive robots, particularly benefit from passive functionalities that require no additional actuators or controllers. Here, we demonstrate that the ERC can provide a systematic method to encode passive body functionalities in a drone platform.

**Fig. 6.**
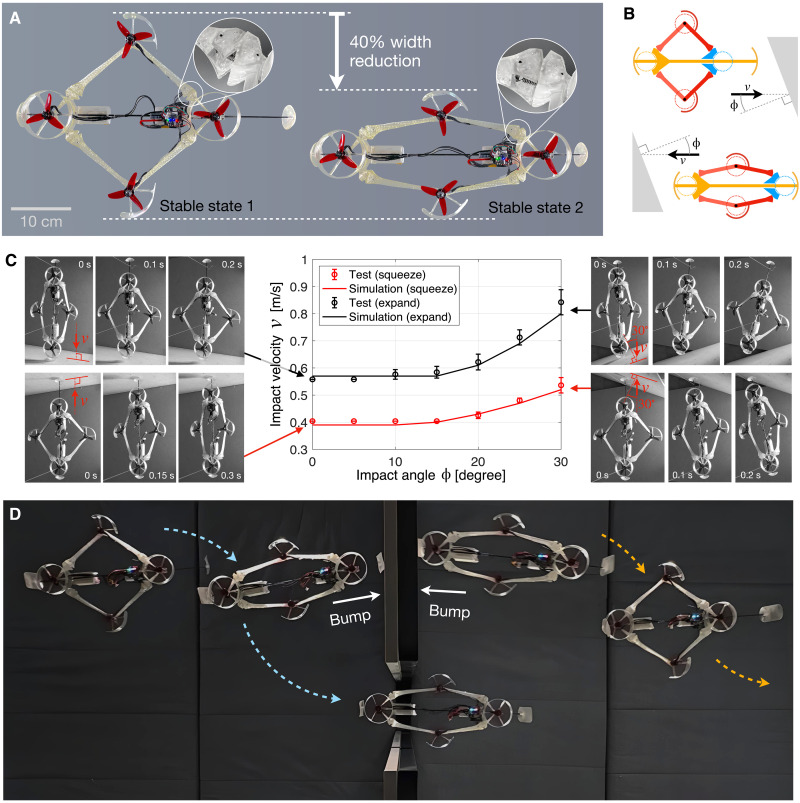
Drone platform with ERC-encoded passive dual-state morphing. (**A**) The ERC elbow joint allows the drone to squeeze by 40%. (**B**) Switching of morphing states by forward (up) and backward (down) bumping. (**C**) Simulation and experiments on the critical impact speed to initiate shape morphing, under different impact angles [defined in (B)], where suspended tests (insets) are repeated seven times at each condition, with the average value and SD (error bar) plotted here. (**D**) Collation of snapshots from flight test (movie S3), demonstrating the morphing toward both directions. This demonstration exemplifies the application of the ERC to encode passive functionalities, including programmable shape transition and collision resilience in weight-sensitive robots.

We designed a quadcopter with a passive dual-state morphing behavior that switches between a standard square configuration and a narrowed/squeezed configuration upon external impact. The behavior is encoded in the bistable stiffness profile of the ERC elbow joint (Fig. 3, bottom). The two morphing states are depicted in Fig. 6A, where the narrowed configuration (state 2) allows for a 40% width reduction, enabling the drone to squeeze through narrow passages, whereas the squared configuration (state 1) allows higher energy efficiency owning to reduced aerodynamic interference between the propellers (*96*). No additional sensors are required to feed back the morphing state to the flight controller as the drone’s symmetrical design naturally maintains the center of mass aligned with the center of thrust. The bistable ERC response is also crucial for stability because the high stiffness near the two stable points helps to prevent oscillations in the morphing angle, which is otherwise inevitable due to torque disturbances from the motor. The drone platform weighs 270 g excluding the batteries. More information on the prototype’s design, manufacturing, and testing can be found in Materials and Methods.

As illustrated in Fig. 6B, upon impact, the blue compartment in the figure slides under its inertia, and the configuration always stabilizes at one of the two stable states determined by the ERC joint’s stiffness profile, thereby realizing the dual-state morphing behavior. Like all bistable systems, switching between the two morphing states requires the compartment’s kinetic energy to overcome the energy barrier resulting from the stiffness response. Therefore, the impact velocity needs to exceed a critical value for morphing to occur. We identified this critical impact velocity through simulation and testing, with results shown in Fig. 6C. The critical velocity differs for the two morphing directions (from the square state to squeezed state, and vice versa) because the residual speed of the compartment after a nonelastic collision depends on the initial morphing state. Details about the simulation and testing are included in Materials and Methods.

The prototype was then flight tested to demonstrate the morphing and narrow passing. A typical operation sequence is shown in Fig. 6D and movie S3, where the drone begins a flight mission in the energy-efficient square configuration, morphs into the squeezed state via a forward bump, thereby pass through the narrow passage. The drone then morphs back to the square state via a backward bump.

This demonstration highlights the potential of the ERC to encode passive morphing in weight-sensitive robots, which particularly benefit from MI-based passive body controls that minimize the number of actuators, thus reducing the mass.

### Lateral compliance

**Fig. 7.**
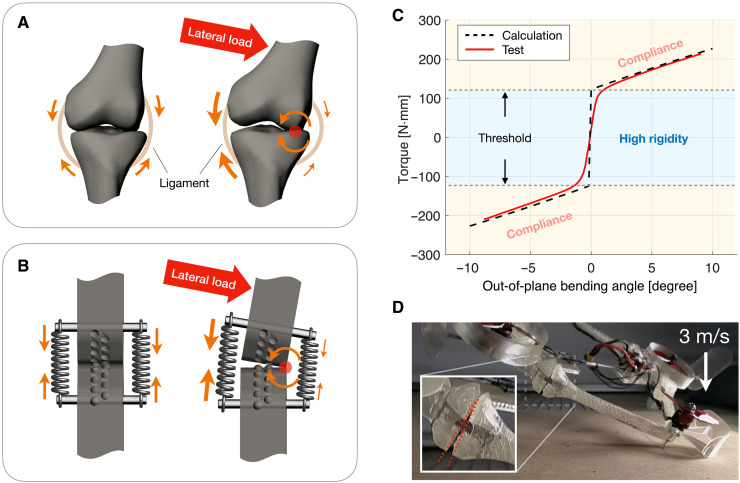
Lateral compliance of ERC. (**A**) Bone-tendon joint with flexible ligaments that realizes lateral deflection through a lateral rolling motion. (**B**) ERC’s lateral compliance in reminiscence of bone-tendon joints. (**C**) Mechanical response of the ERC’s lateral deflection, exhibiting a load-adapting stiffness that reduces upon a torque threshold, which allows load alleviation without sacrificing the initial rigidity under a lower torque. (**D**) Lateral compliance observed in the ERC elbow joint during an impact test (3 m/s) of the dual-state morphing drone prototype.

A distinctive feature that benefits soft robots, particularly in safe human-robot interactions and impact resilience, is the ability of a soft body to provide compliance beyond the primary degree of freedom. The ERC incorporates this feature in a way that resembles bone-tendon joints. As illustrated in Fig. 7A, skeletal joints are usually stabilized by the tension in compliant ligaments that allow lateral deflections (*97*). Similarly, the ERC is laterally stabilized by spring tension, providing a similar lateral compliance as shown in Fig. 7B.

The mechanical characteristic of this lateral compliance is experimentally measured and plotted in Fig. 7C. Similar to the rolling behavior in the primary rotation, the lateral deflection is also realized by a rolling process with a moving contact location. This creates a nonlinear bending response with a torque-adapting stiffness: The joint switches from high stiffness and low compliance to low incremental stiffness and high compliance when the applied lateral torque exceeds a threshold. The value of this threshold depends on the spring tension and the cam’s width. This is ideal for load alleviation because the flexibility is achieved without sacrificing the initial rigidity, which ensures stability and accuracy under nominal load below the torque threshold.

The impact-mitigation behavior is demonstrated using the bistable drone platform, as shown in Fig. 7D, where the lateral deflection of the ERC elbow joint can be observed when the drone impacts the ground at free fall of 3 m/s. It is also worth noting that, under a heavier impact, the ERC exhibits a “dislocation” failure mode, where the cams slide along each other and engage with a different pair of teeth. After dislocation, the rotational stiffness becomes off-design, whereas the structural integrity maintains.

## DISCUSSION

Embodied intelligence, or the physical “encoding” of complex movements and interactions through nonlinear mechanical responses, is widespread in nature. This enables animals to perform challenging tasks with minimal neural or muscular burden, such as the passive undulation of a dead fish swimming upstream (*2*, *3*) or the passive stability in legged animal locomotion (*4*–*9*). To emulate this capability, robots need to implement body components that provide a large number of tunable or “programmable” parameters. The ERC introduced in this study enables accurate programming of complex rotational stiffness profiles, providing a practical pathway toward embodied intelligence.

The potential of a fully programmable, multiparameter rotational stiffness response enabled by the ERC has been demonstrated in a bipedal walker—a classic robotic challenge, and a weight-sensitive morphing drone. These demonstrations highlight the ERC’s capability to encode MI in critical joints of robots to assist challenging tasks or to off-load active control effort to the physical body, as well as to encode passive body functionalities that require no additional actuators or controllers, maintaining simplicity, compactness, light weight, and cost-effectiveness.

It is worth noting that, unlike conventional revolute joints, the ERC rotates through the rolling of cams, thus having no constant rotational axis. Although this introduces complexity in the kinematics, the movement can be accurately calculated based on the cam geometry, and numerical analysis can be carried out, as demonstrated in this study. In addition, although scalability is theoretically possible, and we have successfully produced centimeter and millimeter-scale prototypes, ERCs at other scales may require different materials and manufacturing methods. For instance, submillimeter ERCs will require microscale manufacturing techniques such as two-photon polymerization (*98*) and etching (*99*). Moreover, the springs in the ERCs discussed here are linear elastic, which is typical for common metallic springs and elastomers under relatively low strain. Using nonlinear force-generating devices is also feasible as the ERC’s stiffness response relies on the cam geometry, which can be designed to cope with a nonlinear driving force. This enables designing advanced passive or active ERCs driven by smart-material actuators—e.g. shape memory alloy, artificial muscles, etc.—to realize compact and lightweight systems with responses that are variable on demand, thus actively manipulating the energy of a robotic system, introducing programmable actuating or damping effects.

In conclusion, the ERC is a universally programmable embodiment that combines a minimalist, bone-tendon–inspired compliant design with a scalable cam-based geometry. With verified programmability, scalability, and lateral compliance, it presents a potential solution for endowing robots with embodied control and functionalities.

## MATERIALS AND METHODS

### Statistical analysis of programmability and SF

To assess programmability, we evaluate the mismatch between the simulated ERC response and the randomly generated design target. This mismatch is characterized by the root mean square deviation normalized by the peak torque variation of the target response (i.e., maximum torque − minimum torque). A smaller deviation indicates more accurate programming of the torque response. When the ERC achieves accurate programming across a larger portion of the tested target responses (e.g., with a larger SF), we conclude that the ERC exhibits higher programmability.

To generate Fig. 2C, we simulated 60 million ERC designs with 100,000 randomly generated target responses. Each target response has eight parameters, or five random points along a torque versus rotation angle, with rotation angles between 0° and 90° (the first and the last points have rotation angles of 0° and 90°) and torque between −1 and 1 N·m (normalized torque is used here because torque magnitude linearly scales with the spring’s stiffness and length, thus is theoretically irrelevant to programmability). For each stiffness target, we generate 600 ERC designs across 60 SF values ranging from 1 to 2. For each SF, we generate 10 random combinations of $L_{\text{max}}$, $\Delta L_{\text{max}}$, and $T_{\text{max}}$ as follows: We first assume that ${\text{SF}}_{1}=\text{SF}$, and then a random $L_{\text{max}}$ is generated between 0.001 and 1 m, and $T_{\text{max}}$ is determined using Eq. 1. Then, a random $\Delta L_{\text{max}}$ is generated between $0.1L_{\text{max}}$ and $0.9L_{\text{max}}$, and the energy capacity can be evaluated accordingly: $U_{\text{spring}}=\frac{F_{\text{max}}\cdot \Delta L_{\text{max}}}{2}$; from this, we derive ${\text{SF}}_{2}$ using Eq. 2. If ${\text{SF}}_{2}\geq {\text{SF}}_{1}$, then the parameters are then used to generate the ERC design; otherwise, $T_{\text{max}}$ is scaled up so that ${\text{SF}}_{2}=\text{SF}$. This guarantees that the spring parameters provide the desired unified SF: $\text{SF}=\text{min}\left({\text{SF}}_{1},{\text{SF}}_{2}\right)$.

### ERC prototyping and testing

The centimeter-scale ERCs reported in Fig. 3 are manufactured using a masked stereolithography (MSLA) 3D printer (i.e., a resin printer) with a pixel size under 25 μm, and each ERC is loaded using two metallic springs with $L_{\text{min}}=22$ mm, $L_{\text{max}}=40$ mm, and $T_{\text{max}}=62$ N in total. Each cam has an array of spherical teeth, each with a sphere radius of 1 mm and a height of 0.67 mm. The overall dimension is similar to the illustrations in Fig. 1B.

The scaled-down millimeter-sized ERC reported in Fig. 4 is manufactured using the same 3D printer. Because tension is applied using Kevlar fiber and rubber band, the equivalent spring length equals the distance between the two orifices, whereas the equivalent spring tension and energy corresponds to those of the rubber band, with $L_{\text{min}}=-4$ mm, $L_{\text{max}}=3$ mm, and $T_{\text{max}}=1.8$ N. Note that, in this case, $L_{\text{min}}$ can take a negative value because the contraction of the rubber band is not limited by the distance between the orifices (equivalent spring pivot points). The silicone rubber band naturally has nonlinear elasticity. To reduce this effect thus providing linear elasticity equivalent to metallic springs, we used long rubber bands with excessive $\Delta L_{\text{max}}$, limiting the rubber band’s length variation during operation to below 4% of its original length. The spherical teeth have a radius of 0.4 mm and a height of 0.27 mm.

Tests are conducted using setups similar to three-point bending, which is illustrated in fig. S1 and described in Supplementary Text. The models are loaded using a linear motor at 4 mm/s, and the force responses are measured using a 50-N load cell.

### Bipedal walker simulation and optimization

The bipedal walker is simulated using Matlab Simulink. For simplicity, the knees’ movement is determined by assuming a circular ERC profile. This simplification has negligible effect on the legs’ kinematics as the cam radius variation is lower than 3% in this ERC design. The static and dynamic friction coefficients are 1 and 0.9, respectively. We have chosen high friction coefficients to reduce the sim to reality gap associated with feet slippage. The contact between the foot and ground has a contact stiffness of 50 N/mm and a damping coefficient of 500 N·s/m, which prevent bouncing upon heel strike.

The GA optimization is conducted using the Matlab GA function with default settings, a constant population size of 50, and continued for 50 generations. The initial population is generated with random knee responses, parameterized as illustrated in Fig. 5B, where the parameters take arbitrary values between 0 and 60 N·mm. Higher values lead to insufficient knee flexion and thus are avoided. Each individual is simulated across 70 sets of actuation parameters, with actuation frequencies ranging from 3 to 6 Hz and peak amplitudes ranging from 21° to 30°. The fitness of each individual is determined by the number of frequency-amplitude combinations allowing the robot to walk without falling for at least 6 s.

### Bipedal walker prototyping and testing

The legs are manufactured using the same MSLA 3D printer mentioned above. The hips are directly driven by two KST DS215MG servo motors with a rotation speed of 1200°/s under zero load and 8.4-V power, allowing a maximum harmonic frequency of 6.4 Hz at 30° peak amplitude. The servos are controlled by an Arduino Nano board that generates harmonic Pulse Width Modulation (PWM) signals at the prescribed frequencies and amplitudes. The bottom of the feet are covered with D3O foam with 3-mm thickness, which provides sufficient compliance and damping to prevent bouncing upon heel strike, as well as sufficient friction to prevent feet slippage. The weight of the robot, measured by the static foot pressing force, is 0.27 kg. The test setup is shown in fig. S2 and described in Supplementary Text, where the robot is constrained in the sagittal plane using a rotating boom that allows the robot to pitch. It is worth noting that, although the boom is designed to maximize its bending rigidity while minimizing its weight, oscillations still occur, especially upon heel strike. As a result, there are moments during the experiment when the robot has both feet off the ground, which is not observed in the simulations. During the tests, an Elektro-Automatik EA-PSI 9040-40 T power supply is used to power the robot through a slip ring. The instantaneous power consumption is logged during the tests, where the robot completes three full laps around the boom. The average power consumption and speed are then used to evaluate the COT and Froude number.

### Dual-state morphing drone design, simulation, and testing

As illustrated in Fig. 6B, the drone has a morphing body with one degree of freedom, resembling a parallelogram. The compartment, shown in blue, slides along a Carbon Fiber Reinforced Plastic (CFRP) rod mounted on the yellow compartment. Bearings are used to reduce the sliding friction to ~0.1 N. The flight controller is mounted on the blue compartment, and two identical batteries, each weighing 20% of the drone’s total weight, are installed on the two compartments. This design minimizes variations in the drone’s center of gravity (relative to the center of thrust) during morphing, whereas the compartments maintain a constant orientation, thus reducing inertial disturbances and ensuring steady heading for the flight controller. Structural components of the drone are manufactured using the 3D printer mentioned earlier. Four T-Motor F1507 motors and propellers, with a 3-inch diameter and 4-inch pitch, provide thrust. The prototype weighs 270 g without batteries and 470 g with batteries.

The simulations reported in Fig. 6C are conducted in Matlab Simulink. The friction of compartment sliding is set to 0.1 N according to the measurement. The contact stiffness for the impacts is set to 10 kN/mm. Variation in results is below 2% when the contact stiffness changes by 10 times. No damping is included, considering that the materials are operating in the elastic region.

Test results on critical impact speed for morphing activation (Fig. 6C) are measured using a cable-pendulum setup. The drone is suspended by two nylon cables with 1.5-m length. For the impact test, the drone is raised to a pendulum angle determined by the target impact speed, and impact occurs at the lowest point of the pendulum.

## References

[1] R. Pfeifer, M. Lungarella, F. Iida, Self-organization, embodiment, and biologically inspired robotics. Science 318, 1088–1093 (2007). 18006736 10.1126/science.1145803

[2] D. N. Beal, F. S. Hover, M. S. Triantafyllou, J. C. Liao, G. V. Lauder, Passive propulsion in vortex wakes. J. Fluid Mech. 549, 385–402 (2006).

[3] M. W. Westneat, S. A. Wainwright, 7. Mechanical design for swimming: Muscle, tendon, and bone. Fish Physiol. 19, 271–311 (2001).

[4] M. A. Daley, A. A. Biewener, Running over rough terrain reveals limb control for intrinsic stability. Proc. Natl. Acad. Sci. U.S.A. 103, 15681–15686 (2006). 17032779 10.1073/pnas.0601473103 PMC1622881

[5] D. L. Jindrich, R. J. Full, Dynamic stabilization of rapid hexapedal locomotion. J. Exp. Biol. 205, 2803–2823 (2002). 12177146 10.1242/jeb.205.18.2803

[6] N. U. Schaller, B. Herkner, R. Villa, P. Aerts, The intertarsal joint of the ostrich (*Struthio camelus*): Anatomical examination and function of passive structures in locomotion. J. Anat. 214, 830–847 (2009). 19538629 10.1111/j.1469-7580.2009.01083.x PMC2705294

[7] A. Mo, F. Izzi, E. C. Gönen, D. Haeufle, A. Badri-Spröwitz, Slack-based tunable damping leads to a trade-off between robustness and efficiency in legged locomotion. Sci. Rep. 13, 3290 (2023). 36841875 10.1038/s41598-023-30318-3 PMC9968281

[8] Y.-H. Chang, L. H. Ting, Mechanical evidence that flamingos can support their body on one leg with little active muscular force. Biol. Lett. 13, 20160948 (2017). 28539457 10.1098/rsbl.2016.0948 PMC5454233

[9] H. Geyer, H. Herr, A muscle-reflex model that encodes principles of legged mechanics produces human walking dynamics and muscle activities. IEEE Trans. Neural Syst. Rehabil. Eng. 18, 263–273 (2010). 20378480 10.1109/TNSRE.2010.2047592

[10] H. Hauser, T. Nanayakkara, F. Forni, Leveraging morphological computation for controlling soft robots: Learning from nature to control soft robots. IEEE Control Syst. Mag. 43, 114–129 (2023).

[11] A. Gupta, S. Savarese, S. Ganguli, L. Fei-Fei, Embodied intelligence via learning and evolution. Nat. Commun. 12, 5721 (2021). 34615862 10.1038/s41467-021-25874-z PMC8494941

[12] D. Howard, A. E. Eiben, D. F. Kennedy, J.-B. Mouret, P. Valencia, D. Winkler, Evolving embodied intelligence from materials to machines. Nat. Mach. Intell. 1, 12–19 (2019).

[13] H. Hauser, A. J. Ijspeert, R. M. Füchslin, R. Pfeifer, W. Maass, Towards a theoretical foundation for morphological computation with compliant bodies. Biol. Cybern. 105, 355–370 (2011). 22290137 10.1007/s00422-012-0471-0

[14] H. Hauser, A. J. Ijspeert, R. M. Füchslin, R. Pfeifer, W. Maass, The role of feedback in morphological computation with compliant bodies. Biol. Cybern. 106, 595–613 (2012). 22956025 10.1007/s00422-012-0516-4

[15] A. Carter, W.-H. Chen, S. Misra, C. Sung, *IOP Conference Series: Materials Science and Engineering* (IOP Publishing, 2023), vol. 1292, p. 012003.

[16] S. Collins, A. Ruina, R. Tedrake, M. Wisse, Efficient bipedal robots based on passive-dynamic walkers. Science 307, 1082–1085 (2005). 15718465 10.1126/science.1107799

[17] P. A. Bhounsule, J. Cortell, A. Grewal, B. Hendriksen, J. D. Karssen, C. Paul, A. Ruina, Low-bandwidth reflex-based control for lower power walking: 65 km on a single battery charge. Int. J. Rob. Res. 33, 1305–1321 (2014).

[18] S. Islam, K. Carter, J. Yim, J. Kyle, S. Bergbreiter, A. M. Johnson, in *2022 International Conference on Robotics and Automation (ICRA)* (IEEE, 2022), pp. 207–213.

[19] M. Wisse, J. Van Frankenhuyzen, “Design and construction of MIKE; a 2-D autonomous biped based on passive dynamic walking” in *Adaptive Motion of Animals and Machines* (Springer, 2006), pp. 143–154.

[20] S. H. Collins, M. Wisse, A. Ruina, A three-dimensional passive-dynamic walking robot with two legs and knees. Int. J. Rob. Res. 20, 607–615 (2001).

[21] F. Iida, Y. Minekawa, J. Rummel, A. Seyfarth, Toward a human-like biped robot with compliant legs. Rob. Auton. Syst. 57, 139–144 (2009).

[22] S. Bai, Q. Pan, R. Ding, H. Jia, Z. Yang, P. Chirarattananon, An agile monopedal hopping quadcopter with synergistic hybrid locomotion. Sci. Robot. 9, eadi8912 (2024). 38598611 10.1126/scirobotics.adi8912

[23] F. Iida, R. Pfeifer, Sensing through body dynamics. Rob. Auton. Syst. 54, 631–640 (2006).

[24] J. Tang, K. P. Jain, M. W. Mueller, Quartm: A quadcopter with unactuated rotor tilting mechanism capable of faster, more agile, and more efficient flight. Front. Robot. AI 9, 1033715 (2022). 36340575 10.3389/frobt.2022.1033715 PMC9634085

[25] N. Bucki, J. Tang, M. W. Mueller, Design and control of a midair-reconfigurable quadcopter using unactuated hinges. IEEE Trans. Robot. 39, 539–557 (2022).

[26] A. Badri-Spröwitz, A. Aghamaleki Sarvestani, M. Sitti, M. A. Daley, Birdbot achieves energy-efficient gait with minimal control using avian-inspired leg clutching. Sci. Robot. 7, eabg4055 (2022). 35294220 10.1126/scirobotics.abg4055

[27] F. Corucci, M. Calisti, H. Hauser, C. Laschi, in *Proceedings of the 2015 Annual Conference on Genetic and Evolutionary Computation* (Association for Computing Machinery, 2015), pp. 145–152.

[28] Q. Zhao, K. Nakajima, H. Sumioka, H. Hauser, R. Pfeifer, in *2013 IEEE/RSJ International Conference on Intelligent Robots and Systems* (IEEE, 2013), pp. 1445–1451.

[29] K. Caluwaerts, M. D’Haene, D. Verstraeten, B. Schrauwen, Locomotion without a brain: Physical reservoir computing in tensegrity structures. Artif. Life 19, 35–66 (2013). 23186351 10.1162/ARTL_a_00080

[30] K. Becker, C. Teeple, N. Charles, Y. Jung, D. Baum, J. C. Weaver, L. Mahadevan, R. Wood, Active entanglement enables stochastic, topological grasping. Proc. Natl. Acad. Sci. U.S.A. 119, e2209819119 (2022). 36215466 10.1073/pnas.2209819119 PMC9586297

[31] E. Brown, N. Rodenberg, J. Amend, A. Mozeika, E. Steltz, M. R. Zakin, H. Lipson, H. M. Jaeger, Universal robotic gripper based on the jamming of granular material. Proc. Natl. Acad. Sci. U.S.A. 107, 18809–18814 (2010).

[32] S. Li, J. J. Stampfli, H. J. Xu, E. Malkin, E. V. Diaz, D. Rus, R. J. Wood, in *2019 International Conference on Robotics and Automation (ICRA)* (IEEE, 2019), pp. 7401–7408.

[33] J. Hughes, P. Maiolino, F. Iida, An anthropomorphic soft skeleton hand exploiting conditional models for piano playing. Sci. Robot. 3, eaau3098 (2018). 33141692 10.1126/scirobotics.aau3098

[34] K. Nakajima, H. Hauser, T. Li, R. Pfeifer, Exploiting the dynamics of soft materials for machine learning. Soft Robot. 5, 339–347 (2018). 29708857 10.1089/soro.2017.0075 PMC5995269

[35] Z. Lin, Z. Wang, W. Zhao, Y. Xu, X. Wang, T. Zhang, Z. Sun, L. Lin, Z. Peng, Recent advances in perceptive intelligence for soft robotics. Adv. Intell. Syst. 5, 2200329 (2023).

[36] D. Rus, M. T. Tolley, Design, fabrication and control of soft robots. Nature 521, 467–475 (2015). 26017446 10.1038/nature14543

[37] K. Ghazi-Zahedi, *Morphological Intelligence* (Springer, 2019).

[38] V. Chandiramani, A. T. Conn, H. Hauser, in *IOP Conference Series: Materials Science and Engineering* (IOP Publishing, 2023), vol. 1292, p. 012004.

[39] K. M. Schmoller, A. R. Bausch, Similar nonlinear mechanical responses in hard and soft materials. Nat. Mater. 12, 278–281 (2013). 23511568 10.1038/nmat3603

[40] K. Bertoldi, V. Vitelli, J. Christensen, M. Van Hecke, Flexible mechanical metamaterials. Nat. Rev. Mater. 2, 1–11 (2017).

[41] Q. Chen, K. Tan, X. He, A. Chen, Y. Li, Metastructures based on graded tube inversion for arbitrarily prescribable force-displacement relationships. Extreme Mech. Lett. 70, 102174 (2024).

[42] S. Janbaz, F. Bobbert, M. J. Mirzaali, A. Zadpoor, Ultra-programmable buckling-driven soft cellular mechanisms. Mater. Horiz. 6, 1138–1147 (2019).

[43] A. Rafsanjani, A. Akbarzadeh, D. Pasini, Snapping mechanical metamaterials under tension. Adv. Mater. 27, 5931–5935 (2015). 26314680 10.1002/adma.201502809

[44] R. Wu, P. C. Roberts, S. Lyu, F. Zheng, C. Soutis, C. Diver, D. Zhou, L. Li, Z. Deng, Lightweight self-forming super-elastic mechanical metamaterials with adaptive stiffness. Adv. Funct. Mater. 31, 2008252 (2021).

[45] C. S. Ha, D. Yao, Z. Xu, C. Liu, H. Liu, D. Elkins, M. Kile, V. Deshpande, Z. Kong, M. Bauchy, X. Zheng, Rapid inverse design of metamaterials based on prescribed mechanical behavior through machine learning. Nat. Commun. 14, 5765 (2023). 37718343 10.1038/s41467-023-40854-1 PMC10505607

[46] G. Maloisel, E. Knoop, C. Schumacher, B. Thomaszewski, M. Bächer, S. Coros, Optimal design of flexible-link mechanisms with desired load-displacement profiles. IEEE Robot. Autom. Lett. 8, 4203–4210 (2023).

[47] N. K. Brown, A. P. Garland, G. M. Fadel, G. Li, Deep reinforcement learning for the rapid on-demand design of mechanical metamaterials with targeted nonlinear deformation responses. Eng. Appl. Artif. Intel. 126, 106998 (2023).

[48] B. Deng, A. Zareei, X. Ding, J. C. Weaver, C. H. Rycroft, K. Bertoldi, Inverse design of mechanical metamaterials with target nonlinear response via a neural accelerated evolution strategy. Adv. Mater. 34, e2206238 (2022). 36103610 10.1002/adma.202206238

[49] C. V. Jutte, S. Kota, Design of nonlinear springs for prescribed load-displacement functions. J. Mech. Des. 130, 081403 (2008).

[50] T. E. Bruns, D. A. Tortorelli, Topology optimization of non-linear elastic structures and compliant mechanisms. Comput. Methods Appl. Mech. Eng. 190, 3443–3459 (2001).

[51] D. S. Shah, J. W. Booth, R. L. Baines, K. Wang, M. Vespignani, K. Bekris, R. Kramer-Bottiglio, Tensegrity robotics. Soft Robot. 9, 639–656 (2022). 34705572 10.1089/soro.2020.0170

[52] D. Zappetti, R. Arandes, E. Ajanic, D. Floreano, Variable-stiffness tensegrity spine. Smart Mater. Struct. 29, 075013 (2020).

[53] D. Zappetti, S. H. Jeong, J. Shintake, D. Floreano, Phase changing materials-based variable-stiffness tensegrity structures. Soft Robot. 7, 362–369 (2020). 31851862 10.1089/soro.2019.0091 PMC7301330

[54] D. Zappetti, Y. Sun, M. Gevers, S. Mintchev, D. Floreano, Dual stiffness tensegrity platform for resilient robotics. Adv. Intell. Syst. 4, 2200025 (2022).

[55] H. Lee, Y. Jang, J. K. Choe, S. Lee, H. Song, J. P. Lee, N. Lone, J. Kim, 3D-printed programmable tensegrity for soft robotics. Sci. Robot. 5, eaay9024 (2020). 33022636 10.1126/scirobotics.aay9024

[56] H. Fang, S.-C. A. Chu, Y. Xia, K.-W. Wang, Programmable self-locking origami mechanical metamaterials. Adv. Mater. 30, 1706311 (2018). 10.1002/adma.201706311 29513374

[57] Y. Miyazawa, H. Yasuda, H. Kim, J. H. Lynch, K. Tsujikawa, T. Kunimine, J. R. Raney, J. Yang, Heterogeneous origami-architected materials with variable stiffness. Commun. Mater. 2, 110 (2021).

[58] S.-M. Baek, S. Yim, S.-H. Chae, D.-Y. Lee, K.-J. Cho, Ladybird beetle–inspired compliant origami. Sci. Robot. 5, eaaz6262 (2020). 33022627 10.1126/scirobotics.aaz6262

[59] N. P. Bende, A. A. Evans, S. Innes-Gold, L. A. Marin, I. Cohen, R. C. Hayward, C. D. Santangelo, Geometrically controlled snapping transitions in shells with curved creases. Proc. Natl. Acad. Sci. U.S.A. 112, 11175–11180 (2015). 26294253 10.1073/pnas.1509228112 PMC4568692

[60] K. Liu, T. Tachi, G. H. Paulino, Bio-inspired origami metamaterials with metastable phases through mechanical phase transitions. J. Appl. Mech. 88, 091002 (2021).

[61] B. Liu, J. L. Silverberg, A. A. Evans, C. D. Santangelo, R. J. Lang, T. C. Hull, I. Cohen, Topological kinematics of origami metamaterials. Nat. Phys. 14, 811–815 (2018).

[62] J. L. Silverberg, J.-H. Na, A. A. Evans, B. Liu, T. C. Hull, C. D. Santangelo, R. J. Lang, R. C. Hayward, I. Cohen, Origami structures with a critical transition to bistability arising from hidden degrees of freedom. Nat. Mater. 14, 389–393 (2015). 25751075 10.1038/nmat4232

[63] J. L. Silverberg, A. A. Evans, L. McLeod, R. C. Hayward, T. Hull, C. D. Santangelo, I. Cohen, Using origami design principles to fold reprogrammable mechanical metamaterials. Science 345, 647–650 (2014). 25104381 10.1126/science.1252876

[64] P. Sareh, P. Chermprayong, M. Emmanuelli, H. Nadeem, M. Kovac, Rotorigami: A rotary origami protective system for robotic rotorcraft. Sci. Robot. 3, eaah5228 (2018). 33141756 10.1126/scirobotics.aah5228

[65] S. Mintchev, J. Shintake, D. Floreano, Bioinspired dual-stiffness origami. Sci. Robot. 3, eaau0275 (2018). 33141731 10.1126/scirobotics.aau0275

[66] J. A. Faber, A. F. Arrieta, A. R. Studart, Bioinspired spring origami. Science 359, 1386–1391 (2018). 29567709 10.1126/science.aap7753

[67] W. Kim, J. Byun, J.-K. Kim, W.-Y. Choi, K. Jakobsen, J. Jakobsen, D.-Y. Lee, K.-J. Cho, Bioinspired dual-morphing stretchable origami. Sci. Robot. 4, eaay3493 (2019). 33137780 10.1126/scirobotics.aay3493

[68] W.-H. Chen, W. Yang, L. Peach, D. E. Koditschek, C. R. Sung, Kinegami: Algorithmic design of compliant kinematic chains from tubular origami. IEEE Trans. Robot. 39, 1260–1280 (2023).

[69] R. Wu, L. Li, Z. Wang, Shearigami: Self-folding via shear deformation. Adv. Intell. Syst. 5, 2300020 (2023).

[70] L. Zimmermann, K. Shea, T. Stanković, Conditions for rigid and flat foldability of degree-n vertices in origami. J. Mech. Robot. 12, 011020 (2020).

[71] T. A. Evans, R. J. Lang, S. P. Magleby, L. L. Howell, Rigidly foldable origami gadgets and tessellations. R. Soc. Open Sci. 2, 150067 (2015). 26473037 10.1098/rsos.150067 PMC4593671

[72] L. Lu, S. Leanza, R. R. Zhao, Origami with rotational symmetry: A review on their mechanics and design. Appl. Mech. Rev. 75, 050801 (2023).

[73] K.-H. Hsiao, H.-S. Yan, *Mechanisms in Ancient Chinese Books with Illustrations* (Springer, 2014).

[74] D. D. Swade, Redeeming Charles Babbage’s mechanical computer. Sci. Am. 268, 86–91 (1993).8446883

[75] W. D. Hillis, The Science of Time 2016: Time in Astronomy & Society, Past, Present and Future (Springer, 2017), pp. 331–336.

[76] H. Yasuda, P. R. Buskohl, A. Gillman, T. D. Murphey, S. Stepney, R. A. Vaia, J. R. Raney, Mechanical computing. Nature 598, 39–48 (2021). 34616053 10.1038/s41586-021-03623-y

[77] X. Hu, Z. Song, T. Ma, Novel design method for nonlinear stiffness actuator with user-defined deflection-torque profiles. Mech. Mach. Theory 146, 103712 (2020).

[78] Y. Yao, X. Wang, H. Li, Design and analysis of a high-static-low-dynamic stiffness isolator using the cam-roller-spring mechanism. J. Vib. Acoust. 142, 021009 (2020).

[79] V. Arakelian, Gravity compensation in robotics. Adv. Robot. 30, 79–96 (2016).

[80] J. Medina, P. Lozano, A. Jardón, C. Balaguer, in *2016 IEEE/RSJ International Conference on Intelligent Robots and Systems (IROS)* (IEEE, 2016), pp. 2444–2451.

[81] S. Wolf, G. Grioli, O. Eiberger, W. Friedl, M. Grebenstein, H. Höppner, E. Burdet, D. G. Caldwell, R. Carloni, M. G. Catalano, D. Lefeber, S. Stramigioli, N. Tsagarakis, M. V. Damme, R. V. Ham, B. Vanderborght, L. C. Visser, A. Bicchi, A. Albu-Schaffer, Variable stiffness actuators: Review on design and components. IEEE ASME Trans. Mechatron. 21, 2418–2430 (2015).

[82] N. G. Tsagarakis, I. Sardellitti, D. G. Caldwell, in *2011 IEEE/RSJ International Conference on Intelligent Robots and Systems* (IEEE, 2011), pp. 378–383.

[83] S. Wolf, O. Eiberger, G. Hirzinger, in *2011 IEEE International Conference on Robotics and Automation* (IEEE, 2011), pp. 5082–5089.

[84] S. A. Migliore, E. A. Brown, S. P. DeWeerth, Novel nonlinear elastic actuators for passively controlling robotic joint compliance. J. Mech. Des. 129, 406–412 (2006).

[85] D. R. Carrier, C. S. Gregersen, N. A. Silverton, Dynamic gearing in running dogs. J. Exp. Biol. 201, 3185–3195 (1998). 9808832 10.1242/jeb.201.23.3185

[86] W. M. Murray, T. S. Buchanan, S. L. Delp, Scaling of peak moment arms of elbow muscles with upper extremity bone dimensions. J. Biomech. 35, 19–26 (2002). 11747879 10.1016/s0021-9290(01)00173-7

[87] M. G. Wheatley, D. G. Thelen, K. J. Deluzio, M. J. Rainbow, Knee extension moment arm variations relate to mechanical function in walking and running. J. R. Soc. Interface 18, 20210326 (2021). 34404228 10.1098/rsif.2021.0326 PMC8371375

[88] A. Navacchia, V. Kefala, K. B. Shelburne, Dependence of muscle moment arms on in vivo three-dimensional kinematics of the knee. Ann. Biomed. Eng. 45, 789–798 (2017). 27620064 10.1007/s10439-016-1728-x PMC5411230

[89] J. M. Jani, M. Leary, A. Subic, M. A. Gibson, A review of shape memory alloy research, applications and opportunities. Mater. Des. 56, 1078–1113 (2014).

[90] L. R. Meza, S. Das, J. R. Greer, Strong, lightweight, and recoverable three-dimensional ceramic nanolattices. Science 345, 1322–1326 (2014). 25214624 10.1126/science.1255908

[91] L. Girardi, G. Risso, L. Pesaresi, P. Ermanni, S. Mintchev, Multistable composite laminate grids as a design tool for soft reconfigurable multirotors. Adv. Intell. Syst. 2024, 2400356 (2024).

[92] M. Zhao, K. Okada, M. Inaba, Versatile articulated aerial robot dragon: Aerial manipulation and grasping by vectorable thrust control. Int. J. Rob. Res. 42, 214–248 (2023).

[93] F. Ruiz, B. C. Arrue, A. Ollero, Sophie: Soft and flexible aerial vehicle for physical interaction with the environment. IEEE Robot. Autom. Lett. 7, 11086–11093 (2022).

[94] A. Fabris, E. Aucone, S. Mintchev, Crash 2 squash: An autonomous drone for the traversal of narrow passageways. Adv. Intell. Syst. 4, 2200113 (2022).

[95] D. Falanga, K. Kleber, S. Mintchev, D. Floreano, D. Scaramuzza, The foldable drone: A morphing quadrotor that can squeeze and fly. IEEE Robot. Autom. Lett. 4, 209–216 (2018).

[96] S. Dougherty, N. L. Oo, D. Zhao, Effects of propeller separation and onset flow condition on the performance of quadcopter propellers. Aerosp. Sci. Technol. 145, 108837 (2024).

[97] K. L. Markolf, J. Mensch, H. C. Amstutz, Stiffness and laxity of the knee—The contributions of the supporting structures. A quantitative in vitro study. JBJS 58, 583–594 (1976). 946969

[98] C. Hong, Y. Wu, C. Wang, Z. Ren, C. Wang, Z. Liu, W. Hu, M. Sitti, Wireless flow-powered miniature robot capable of traversing tubular structures. Sci. Robot. 9, eadi5155 (2024). 38478591 10.1126/scirobotics.adi5155

[99] Y. Chen, H. Wang, E. F. Helbling, N. T. Jafferis, R. Zufferey, A. Ong, K. Ma, N. Gravish, P. Chirarattananon, M. Kovac, R. J. Wood, A biologically inspired, flapping-wing, hybrid aerial-aquatic microrobot. Sci. Robot. 2, eaao5619 (2017). 33157886 10.1126/scirobotics.aao5619

