# Supplementary material for: Encoding mechanical intelligence using ultraprogrammable joints
Source: Sci Adv. 2025 Apr 23;11(17):eadv2052. doi: 10.1126/sciadv.adv2052 (PMC12017317; doi:10.1126/sciadv.adv2052)
Supplement: Supplementary file 1 — Figs. S1 to S3 Supplementary Text Legends for movies S1 to S3 [file sciadv.adv2052_sm.pdf]

Science Advances
AAAS

Supplementary Materials for

Encoding mechanical intelligence using ultraprogrammable joints

Rui Wu *et al.*

Corresponding author: Rui Wu, rui.wu@bristol.ac.uk

Sci. Adv. **11**, eadv2052 (2025)
DOI: 10.1126/sciadv.adv2052

The PDF file includes:

Figs. S1 to S3
Supplementary Text
Legends for movies S1 to S3

Other Supplementary Material for this manuscript includes the following:

Movies S1 to S3

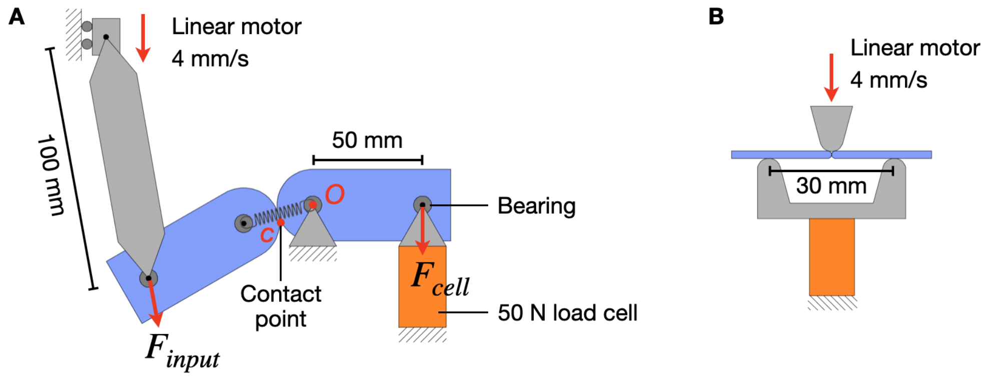

Figure S1: **ERC torque response measurement setup.** (**A**) Setup for the centimeter-sized models. (**B**) Setup for the millimeter-sized models.

Supplementary Text

Torque response measurement

The centimeter-sized models are tested using a setup illustrated in Fig. S1A. The right-side cam of the ERC is constrained by a ground support and a 50 N load cell. The left side cam is loaded using a linear motor at 4 mm/s. All pivots use bearings to minimise friction. Analysing the entire ERC with respect to pivot O, we can evaluate the force F_{input}, which balances the torque produced by the measured force F_{cell}, using simple geometry. Then, the left-side cam is analysed with respect to the cams contacting point C, where the torque produced by F_{input} balances the joint bending torque. The joint response is thereby evaluated from the force measurement F_{cell}, and the joint angle is evaluated from the linear motor displacement using geometry.

The millimeter-sized models are tested using a typical 3-point-bending setup illustrated in Fig. S1B.

Bipedal walker prototyping and testing

The bipedal walker prototype is shown in Fig. S2A. In the walking test setup shown in Fig. S2B, the robot is constrained in the sagittal plane using a rotating boom. The boom has a main beam made of Carbon Fibre Reinforced Plastic (CFRP) rods with a 6 mm by 6 mm square cross-section, and secondary trusses made from CFRP rods with a 3 mm diameter. The secondary trusses are added to reduce the boom oscillation. Ballast mass is used to replicate the simulated angular

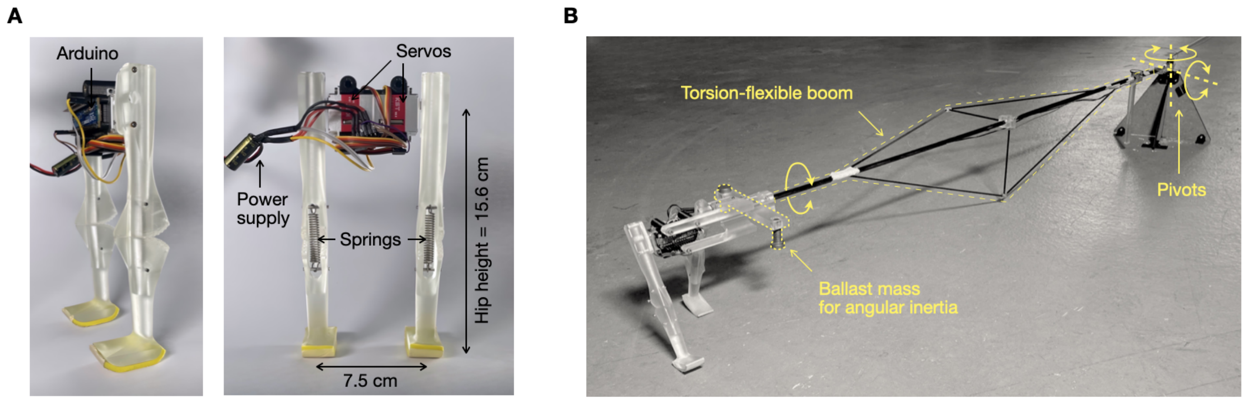

Figure S2: **Bipedal walker test setup.** (**A**) Design of the prototype. (**B**) Walking test setup.

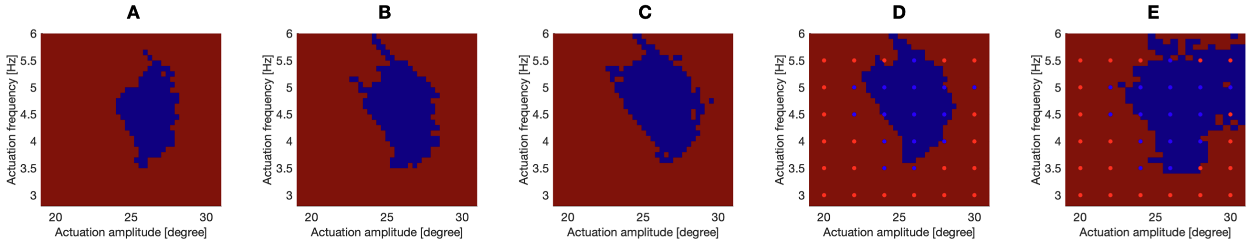

Figure S3: **Stability maps with different knee responses and setup.** (**A**) 3-parameter knee response. (**B**) 4-parameter knee response. (**C**) 5-parameter knee response. (**D**) 6-parameter knee response, overlaid with test results. (**E**) 6-parameter knee response with 0.002 N·m/°pitch stabilisation effect. (blue: stable; red: unstable)

inertia and weight. The boom has a weak torsional stiffness of approximately 0.002 N·m/°, which provides a weak pitch-stabilising torque for the robot. This weak stabilisation helps reducing the disturbances caused by mechanical backlash of the servos, and structural oscillation of the boom. In the simulation, the weak pitching constraint has notable effect only in the high actuation amplitude region, as shown by the simulation results in Fig. S3E, indicating that the test results can effectively reflect the designed stability.

Caption for Movie S1. Four basic types of rotational stiffness achieved by the ERC. Animated version of Fig. 1B.

Caption for Movie S2. Bipedal walking encoded by ERC knee joint. The prototype walks across steps, and achieves stable walking at a range of harmonic hip actuation.

Caption for Movie S3. Quadcopter with ERC-encoded passive dual-state morphing. Animated version of Fig. 6D.
